# Supplementary material for: Defining dual-axis landscape gradients of human influence for studying ecological processes
Source: PLoS One. 2021 Nov 18;16(11):e0252364. doi: 10.1371/journal.pone.0252364 (PMC8601559; doi:10.1371/journal.pone.0252364)
Supplement: S1 File — (DOCX) [file pone.0252364.s001.docx]

S1

Supplemental Landscape Gradient Analysis

*Effects spatial extent: 30 x 30 km city window*

To test for sensitivity of our landscape quantification approach to the spatial extent (e.g., window) surrounding each city, we conducted the same analysis for a 30*x*30 km window centered on each city. As anticipated, results for the 30*x*30 km extent were consistent with those seen at 50*x*50 km (S1 Table 2). Two principal components were retained according to the broken stick cut-off value of 22.1% variance. As with our 50*x*50 km analysis, the first principal component described a gradient between predominantly urbanized – *hard* – landscapes (positive loadings), and a combination of forested, herbaceous and agricultural – *soft* – regions (negative loadings). Therefore, though the direction of the positive and negative loadings was reversed, the ecological interpretation of the resulting landscape gradient remains the same. Similarly, component two described variation between types of *soft* landscapes along a *brown*-to-*green* gradient, with forests and wetlands exhibiting strong negative loadings while croplands and grasslands were strongly positive. This analysis shows that though the direction and precise magnitude of principal components was variable, the ultimate interpretation remains is not impacted by changing spatial extent of analysis.

S1 Table 1: Principal component loadings for a 30x30 km extent surrounding each city and the 50x50 km extent used in the main text (provided for reference). Despite slight variation in the exact magnitude and direction of loadings, changing spatial extent surrounding each city both resulted in two dominant components describing hard-to-soft and brown-to-green landscape gradients, respectively.

| NLCD Layer | | Obs. Freq. | PC 1 | | PC2 | | PC3 | |
| --- | --- | --- | --- | --- | --- | --- | --- | --- |
| *City Extent* | |  | 50 km | 30 km | 50 km | 30 km | 50 km | 30 km |
| *Std. Dev.* | |  | 1.581 | 1.760 | 1.295 | 1.445 | 1.182 | 1.234 |
| *Variance Explained (%)* | |  | 16.7 | 20.7 | 11.1 | 13.9 | 9.3 | 11.7 |
| *Water* | 11 - Open Water | 2.70 % | 0.042 | -0.029 | 0.030 | -**0.238** | -0.04 | **0.558** |
| *Developed* | 21 - Devel-Open | 6.49 % | **-0.360** | **0.343** | 0.055 | -0.099 | 0.017 | *-0.204* |
|  | 22 - Devel-Low | 6.72 % | **-0.545** | **0.512** | 0.047 | 0.005 | -0.040 | -0.020 |
|  | 23 - Devel-Mid | 4.78 % | **-0.553** | **0.513** | 0.015 | 0.0467 | -0.125 | 0.068 |
|  | 24 - Devel-High | 1.61 % | **-0.392** | **0.444** | -0.007 | 0.027 | -0.148 | 0.134 |
| *Barren* | 31 - Barren | 0.79 % | 0.039 | -0.059 | -0.017 | -0.068 | -0.116 | **0.441** |
| *Forest* | 41 - Forest-Deciduous | 10.17 % | *0.119* | -*0.141* | **0.469** | **-0.459** | 0.171 | *-0.187* |
|  | 42 - Forest-Evergreen | 8.96 % | *0.154* | -*0.169* | 0.269 | *-0.141* | **-0.349** | -0.114 |
|  | 43 - Forest-Mixed | 2.14 % | 0.087 | -0.031 | **0.433** | **-0.404** | 0.001 | *-0.198* |
| *Shrubland* | 52 - Scrub / Shrub | 10.56 % | *0.151* | *-0.171* | -0.012 | 0.079 | **-0.554** | 0.097 |
| *Herbaceous* | 71 -Grass / Herb | 10.85 % | *0.140* | *-0.184* | **-0.339** | **0.347** | -0.282 | -0.001 |
| *Cultivated* | 81 - Pasture / Hay | 11.25 % | 0.053 | -0.084 | 0.082 | -0.092 | **0.446** | -0.107 |
|  | 82 - Crop / Cultivated | 19.41 % | *0.119* | *-0.178* | **-0.491** | **0.389** | **0.387** | **-**0.048 |
| *Wetlands* | 90 - Woody Wetl. | 2.81 % | 0.043 | -0.029 | **0.378** | **-0.463** | 0.157 | *-0.113* |
|  | 95 - Herbaceous Wetl. | 0.77 % | 0.032 | -0.051 | 0.075 | -0.175 | -0.039 | 0.**553** |

*Effects of smoothing scale: 1,500-m scale*

To test for sensitivity of landscape quantification approach to choice of smoothing scale, we conducted landscape analysis at two kernel scales, 500-m and 1,500-m described here. Extending the size of the smoothing parameter increases the “neighborhood” surrounding each focal pixel influencing the smoothed value. In this way, a larger portion of the surrounding landscape is considered to have a significant contribution on the smoothed value of a given pixel. We analyzed all ten study cities in a joint analysis at a 1,500-m smoothing scale using the same methods described for 500-m.

Quantification of landscape heterogeneity at a 1,500-m smoothing scale yielded results that were highly comparable with the 500-m scale (S1 Table 2). The first principal component explaining the largest proportion of data variation (σ_1500_ = 19.2%) was also strongly negative for developed land-cover classes (e.g, Devel-Mid = -0.531), with neutral or positive loadings for forested (Forest-Deciduous = 0.034), open, and agricultural (Crop = 0.133) classes. The second principal component (σ_1500_ = 12.9%) showed a strong divergence between non-impervious (i.e., *soft*) landscape types, differentiating between those that are highly modified by human activity. Forested regions had strongly negative loadings (Forest-Deciduous = -0.481) while croplands had strongly positive loadings (Crop = 0.424), and distinguishes between types of non-structural landscapes. Finally, as in the 500-m analysis, the third principal component also distinguished between non-structural landscapes. However, while PC2 separated predominantly deciduous forests and wetlands (common in eastern US) from agriculture, PC3 separated mixed forests and scrublands (common in western US) from agriculture.

While the component weights and NLCD land-use eigen values differed between 500-m and 1,500-m analyses, the ultimate ecological interpretation of the resulting landscape gradients was consistent. In both cases the first component represented a gradient from heavily built to non-built environments along *hard*-to-*soft*, while the second and third represented variation in the non-built environment along an axis of *green*-to-*brown*.

*Effects of local environment: city specific analysis*

In addition to our joint (i.e., all cities) analyses, we quantified landscape gradients in all study cities independently (S1 Table 1). Results for city specific analyses were remarkably consistent in spite of regional variation in the composition and configuration of natural and human-dominated landscapes. This was especially true in the case of the first principal component describing the most significant portion of the variation in landscape data. In all cities the strongest component described a gradient of variation from *hard* regions characterized by the human-dominated built environment from non-built *soft* regions (S1 Table 1). The second component, however, was more variable and depended on the city specific landscape composition. Nevertheless, with the sole exception of Albuquerque, where PC2 differentiated wetland habitats from developed areas, the interpretation of the second component consistently described variation between *brown* and *green* regions, respectively.

S1 Table 2: Principal component loadings for the 1,500-m and 500-m analyses (provided for reference). Analysis at both smoothing scales resulted in hard-to-soft and brown-to-green landscape gradients.

| NLCD Layer | | Obs. Freq. | PC 1 | | PC2 | | PC3 | |
| --- | --- | --- | --- | --- | --- | --- | --- | --- |
| *Smoothing Scale* | |  | 500m | 1500m | 500m | 1500m | 500m | 1500m |
| *Std. Dev.* | |  | 1.581 | 1.695 | 1.295 | 1.388 | 1.182 | 1.219 |
| *Variance Explained (%)* | |  | 16.7 | 19.2 | 11.1 | 12.9 | 9.3 | 9.9 |
| *Water* | 11 - Open Water | 2.70 % | 0.042 | 0.032 | 0.030 | -0.064 | -0.04 | 0.295 |
| *Developed* | 21 - Devel-Open | 6.49 % | **-0.360** | **-0.385** | 0.055 | -0.064 | 0.017 | -0.201 |
|  | 22 - Devel-Low | 6.72 % | **-0.545** | **-0.531** | 0.047 | 0.001 | -0.040 | 0.049 |
|  | 23 - Devel-Mid | 4.78 % | **-0.553** | **-0.531** | 0.015 | 0.042 | -0.125 | 0.122 |
|  | 24 - Devel-High | 1.61 % | **-0.392** | **-0.432** | -0.007 | 0.049 | -0.148 | 0.155 |
| *Barren* | 31 - Barren | 0.79 % | 0.039 | 0.046 | -0.017 | 0.006 | -0.116 | 0.270 |
| *Forest* | 41 - Forest-Deciduous | 10.17 % | *0.119* | 0.034 | **0.469** | **-0.481** | 0.171 | -0.147 |
|  | 42 - Forest-Evergreen | 8.96 % | *0.154* | *0.145* | 0.269 | -0.211 | **-0.349** | **0.302** |
|  | 43 - Forest-Mixed | 2.14 % | 0.087 | 0.047 | **0.433** | **-0.434** | 0.001 | -0.049 |
| *Shrubland* | 52 - Scrub / Shrub | 10.56 % | *0.151* | *0.147* | -0.012 | 0.037 | **-0.554** | **0.465** |
| *Herbaceous* | 71 -Grass / Herb | 10.85 % | *0.140* | *0.159* | **-0.339** | **0.341** | -0.282 | 0.131 |
| *Cultivated* | 81 - Pasture / Hay | 11.25 % | 0.053 | 0.033 | 0.082 | -0.131 | **0.446** | **-0.406** |
|  | 82 - Crop / Cultivated | 19.41 % | *0.119* | *0.133* | **-0.491** | **0.424** | **0.387** | **-0.390** |
| *Wetlands* | 90 - Woody Wetl. | 2.81 % | 0.043 | -0.005 | **0.378** | **-0.439** | 0.157 | -0.089 |
|  | 95 - Herbaceous Wetl. | 0.77 % | 0.032 | 0.019 | 0.075 | -0.88 | -0.039 | 0.281 |

S1 Table 3: City specific analysis of landscape heterogeneity revealed remarkable consistency in spite of significant regional variation in composition and configuration of the natural and human-dominated landscapes. Component one (PC1) clearly differentiated 'urban’ from non-urban regions in all cases. While Component two (PC2) was slightly more variable, in general it described a gradient between types of non-urban habitat (i.e., brown vs. green).

|  | Lincoln | Lincoln | Lubbock | Lubbock | Lexington | Lexington | Worcester | Worcester | Spokane | Spokane |
| --- | --- | --- | --- | --- | --- | --- | --- | --- | --- | --- |
|  | PC1 | PC2 | PC1 | PC2 | PC1 | PC2 | PC1 | PC2 | PC1 | PC2 |
| Open.Water | 0.0097 | -0.1881 | 0.1672 | -0.1530 | 0.0626 | -0.2695 | 0.0192 | -0.0623 | 0.0708 | -0.2448 |
| Devel.Open | **0.3825** | -0.0614 | **0.3206** | 0.1585 | **-0.4375** | -0.0133 | **0.3803** | 0.0477 | **-0.3822** | -0.0028 |
| Devel.Low | **0.4830** | -0.0755 | **0.4564** | 0.2025 | **-0.4892** | -0.1069 | **0.4697** | 0.1046 | **-0.5013** | -0.1937 |
| Devel.Med | **0.5156** | -0.0707 | **0.4653** | 0.2182 | **-0.4947** | -0.1094 | **0.4811** | 0.0248 | **-0.4936** | -0.2196 |
| Devel.High | **0.4144** | -0.0549 | **0.4113** | 0.1829 | **-0.3917** | -0.0876 | **0.3633** | -0.0076 | **-0.3759** | -0.1930 |
| Barren | 0.0461 | 0.0974 | 0.0249 | -0.0502 | -0.0165 | -0.0219 | -0.0256 | 0.3041 | -0.0092 | -0.1043 |
| For.Decid | -0.2193 | **-0.3932** | 0.1444 | **-0.4658** | 0.1874 | -0.4700 | -0.2924 | **-0.4347** | -0.0168 | 0.1634 |
| For.Everg | -0.0504 | -0.1790 | 0.1264 | -0.4005 | 0.1118 | **-0.3514** | -0.2107 | 0.1938 | 0.2803 | **-0.2564** |
| For.Mixed | -0.0939 | -0.2636 | 0.0818 | -0.0887 | 0.1392 | **-0.3639** | -0.2543 | 0.0148 | 0.0108 | 0.1156 |
| Scrub.Shrub | -0.0565 | -0.1232 | 0.0000 | 0.0000 | 0.0878 | -0.0812 | -0.1162 | **0.4301** | 0.2594 | **-0.3358** |
| Grass.Herb | -0.2089 | **-0.5197** | 0.0918 | **-0.4197** | 0.1302 | -0.3872 | -0.0396 | **0.4064** | 0.1722 | -0.1114 |
| Pastr.Hay | -0.1200 | -0.1920 | 0.0872 | -0.3211 | 0.2609 | **0.4560** | -0.1675 | 0.1491 | -0.0038 | -0.0442 |
| Crop.Cultv | -0.2389 | **0.5991** | -0.4523 | **0.1477** | 0.0313 | 0.1001 | -0.0711 | **0.3637** | -0.0260 | **0.6670** |
| Wetl.Wood | 0.0521 | 0.0193 | 0.0977 | -0.3713 | 0.0726 | -0.1982 | -0.1621 | 0.2697 | 0.1211 | -0.2907 |
| Wetld.Herb | 0.0130 | 0.0044 | -0.0307 | -0.0514 | 0.0307 | -0.0713 | 0.0363 | 0.2926 | 0.1464 | -0.2095 |

|  | Salt Lake City | Salt Lake City | Portland | Portland | Albuquer. | Albuquer. | Bakers. | Bakers. | Jackson | Jackson |
| --- | --- | --- | --- | --- | --- | --- | --- | --- | --- | --- |
|  | PC1 | PC2 | PC1 | PC2 | PC1 | PC2 | PC1 | PC2 | PC1 | PC2 |
| Open.Water | -0.0717 | 0.2931 | 0.0792 | -0.3052 | 0.2163 | **-0.4972** | 0.0193 | -0.0229 | 0.0388 | 0.2430 |
| Devel.Open | **0.4247** | -0.1160 | 0.1064 | 0.2453 | **0.3161** | 0.0420 | **-0.3070** | 0.0159 | **0.3806** | -0.2025 |
| Devel.Low | **0.4660** | -0.1832 | **0.3897** | 0.2890 | **0.4762** | 0.1997 | **-0.5122** | 0.2297 | **0.4384** | -0.1857 |
| Devel.Med | **0.4825** | -0.1912 | **0.4092** | 0.2339 | **0.4612** | 0.3012 | **-0.4940** | 0.2853 | **0.4354** | -0.1508 |
| Devel.High | **0.3206** | -0.1323 | **0.3020** | 0.0947 | **0.3882** | 0.2714 | **-0.3921** | 0.2543 | **0.3617** | -0.1210 |
| Barren | -0.0739 | 0.1867 | 0.0350 | **-0.3402** | -0.0230 | -0.0413 | -0.0333 | 0.0141 | -0.0116 | -0.0125 |
| For.Decid | -0.2997 | **-0.3827** | -0.2801 | 0.1769 | -0.0358 | -0.0025 | 0.1978 | **0.3166** | -0.2990 | -0.2695 |
| For.Everg | -0.2748 | **-0.4040** | **-0.3772** | 0.1764 | -0.0841 | 0.0010 | 0.2062 | **0.3595** | -0.1908 | -0.2362 |
| For.Mixed | -0.1726 | **-0.3454** | -0.3648 | 0.1974 | -0.0263 | -0.0019 | 0.1467 | 0.2754 | -0.2506 | **-0.4811** |
| Scrub.Shrub | -0.1590 | 0.2191 | -0.3417 | 0.1162 | -0.3120 | 0.0571 | 0.2615 | **0.3732** | -0.3123 | -0.2486 |
| Grass.Herb | -0.0271 | 0.2500 | -0.2902 | 0.0427 | -0.2284 | 0.0028 | 0.2632 | 0.2326 | -0.1280 | -0.1619 |
| Pastr.Hay | 0.1237 | **0.3233** | -0.1094 | -0.2003 | 0.2280 | **-0.4017** | -0.0112 | -0.2535 | -0.1768 | 0.2425 |
| Crop.Cultv | 0.0836 | 0.1590 | -0.0386 | **-0.2915** | 0.0913 | -0.2729 | -0.0504 | **-0.4830** | -0.0723 | **0.3504** |
| Wetl.Wood | 0.1125 | 0.1092 | 0.0002 | **-0.4278** | 0.2087 | **-0.5238** | 0.0234 | 0.0376 | -0.0247 | **0.3786** |
| Wetld.Herb | -0.0167 | **0.3113** | 0.0498 | **-0.3987** | 0.0105 | -0.1777 | -0.0009 | -0.0510 | -0.0371 | 0.2345 |
