# Supplementary material for: Defining dual-axis landscape gradients of human influence for studying ecological processes
Source: PLoS One. 2021 Nov 18;16(11):e0252364. doi: 10.1371/journal.pone.0252364 (PMC8601559; doi:10.1371/journal.pone.0252364)
Supplement: S2 File — (DOCX) [file pone.0252364.s002.docx]

S2:

American Robin Occupancy Analysis: Supplementary Tables and Figures

S2 Table 1: Total number and final number of sampling locations for each study city. In cases where cities had > 250 locations, we rarefied the sampling pool with a random sample of 250 locations. Number of sampling events reflects the total number of visits to all sampling locations in a given city, and Frequency of Presence is the proportion of those visits where a Robin was observed.

|  | Bakersfield, California | Lexington, Kentucky | Worcester, Massachusetts | Jackson, Mississippi | Lincoln, Nebraska | Albuquerque, NM | Portland,  OR | Lubbock,  TX | Salt Lake City, UT | Spokane,  WA |
| --- | --- | --- | --- | --- | --- | --- | --- | --- | --- | --- |
| Total Num. Sampling Locations | 31 | 181 | **593** | 56 | 165 | **581** | **831** | 46 | **581** | 224 |
| Final Num. Sampling Locations | 31 | 181 | 250 | 56 | 165 | 250 | 250 | 46 | 250 | 224 |
| Final Num. Sampling Events | 110 | 632 | 791 | 200 | 596 | 853 | 869 | 158 | 830 | 740 |
| Freq. of ‘Presence’ | 0.4818 | 0.4430 | 0.4349 | 0.4650 | 0.4463 | 0.4431 | 0.4325 | 0.4620 | 0.4289 | 0.3865 |

S2 Table 2: Full list of candidate models for detection (left) and occupancy (right). All detection models were fit with fit with the fully parameterized detection model ( ~ city * HS * BG) for a total of 26 candidate models. Finally, 16 candidate models for occupancy were considered using the best fit detection model (city*date+date^2^+HS+BG). Here, HS refers to the hard-to-soft gradient, while BG denotes brown-to-green.

| Detection Model Structure | Detection Model Structure |
| --- | --- |
| ~ *1 ~ Ψ* | ~ *p* ~ 1 |
| *~ city ~ Ψ* | ~ *p* ~ *city* |
| ~ *HS ~ Ψ* | ~ *p* ~ *HS* |
| ~ *BG ~ Ψ* | ~ *p* ~ *BG* |
| ~ *date ~ Ψ* | ~ *p* ~ *city+HS* |
| ~ *date + date*^2^  *~ Ψ* | ~ *p* ~ *city+BG* |
| *~ city + date ~ Ψ* | ~ *p* ~ *city*HS* |
| *~ city * date ~ Ψ* | ~ *p* ~ *city*BG* |
| ~ *city*date+ date*^2^ *~ Ψ* | ~ *p* ~ *HS + BG* |
| ~ *city+date+date^2^ ~ Ψ* | ~ *p* ~ *HS * BG* |
| ~ *city*date +BG ~ Ψ* | ~ *p* ~ *city*HS+BG* |
| ~ *city*date+HS ~ Ψ* | ~ *p* ~ *city*BG+HS* |
| ~ *city*date+HS+BG ~ Ψ* | ~ *p* ~ *city+ HS*BG* |
| ~ *city*HS ~ Ψ* | ~ *p* ~ *city*(HS+BG)* |
| ~ *city*BG ~ Ψ* | ~ *p* ~ *city*(HS*BG)* |
| ~ *city*(HS+BG) ~ Ψ* | ~ *p* ~ *city + HS + BG* |
| ~ *city + HS ~ Ψ* |  |
| ~ *city* + *BG ~ Ψ* |  |
| ~ *city + HS + BG ~ Ψ* |  |
| ~ *city * BG + HS + date ~ Ψ* |  |
| ~ *city * HS + BG + date ~ Ψ* |  |
| ~ *city + date + HS + BG. ~ Ψ* |  |
| ~ *city*(date+date^2^) + HS + BG ~ Ψ* |  |
| ~ *city * BG + HS + date + date^2^  ~ Ψ* |  |
| ~ *city * HS + BG + date + date^2^  ~ Ψ* |  |
| ~ *city + HS + BH + date + date^2^  ~ Ψ* |  |

S2 Table 3: Parameter estimates for Robin detection probability (ρ) and occupancy (Ψ) for the best supported model: $logit\left( \rho_{ij} \right)={city}_{i}*{date}_{ij}+{date}_{ij}^{2} {+ HS}_{i}+{BG}_{i}$ and for occupancy, $logit(\psi_{i}) = {city}_{i}{*HS}_{i}+{BG}_{i}$ . All parameter estimates are on the logit scale. State abbreviations are used here for brevity, and reflect the following: KY = Lexington, Kentucky, MA = Worcester, Massachusetts, MS = Jackson, Mississippi, NE = Lincoln, Nebraska, NM = Albuquerque, New Mexico, OR = Portland, Oregon, TX = Lubbock, Texas, UT = Salt Lake City, Utah, WA = Spokane, Washington, and the intercept represents Bakersfield, California.

| Parameter (*p)* | Estimate | Std. Error | p-value | Parameter (Ψ) | Estimate | Std. Error | p-value |
| --- | --- | --- | --- | --- | --- | --- | --- |
| *(Intercept)* | 0.5437 | 1.077 | 0.614 | ***(Intercept)*** | 0.6216 | 0.959 | 0.517 |
| *city.KY* | 0.9950 | 1.099 | 0.365 | ***city.KY*** | 0.5677 | 0.993 | 0.568 |
| *city.MA* | 0.0150 | 1.087 | 0.989 | ***city.MA*** | 1.8075 | 1.186 | 0.127 |
| *city.MS* | -1.4501 | 1.116 | 0.194 | ***city.MS*** | 3.5543 | 7.334 | 0.628 |
| *city.NE* | 1.2558 | 1.097 | 0.252 | ***city.NE*** | 0.7465 | 0.992 | 0.452 |
| *city.NM* | -0.4088 | 1.127 | 0.717 | ***city.NM*** | -0.8356 | 1.009 | 0.408 |
| *city.OR* | -0.8127 | 1.082 | 0.453 | ***city.OR*** | 1.1564 | 1.138 | 0.309 |
| *city.TX* | -1.4047 | 1.114 | 0.207 | ***city.TX*** | 0.4815 | 1.072 | 0.653 |
| *city.UT* | -0.0671 | 1.082 | 0.950 | ***city.UT*** | 0.6174 | 1.017 | 0.545 |
| *city.WA* | -0.148 | 1.094 | 0.989 | ***city.WA*** | 0.9677 | 1.077 | 0.369 |
| *date* | -0.0187 | 0.113 | 0.869 | ***BG*** | 0.5176 | 0.143 | *0.00029* |
| *date^2^* | -0.0122 | 0.0015 | *6.7e^-17^* | ***HS*** | 0.0394 | 0.571 | 0.945 |
| *BG* | 0.1421 | 0.0528 | *0.007* | ***city.KY:HS*** | 0.2868 | 0.640 | 0.654 |
| *HS* | -0.0379 | 0.0422 | 0.369 | ***city.MA:HS*** | -1.7202 | 0.765 | 0.025 |
| *city.KY:date* | 0.1160 | 0.112 | 0.302 | ***city.MS:HS*** | -3.8444 | 5.004 | 0.442 |
| *city.MA:date* | 0.0678 | 0.112 | 0.547 | ***city.NE:HS*** | -0.0307 | 0.594 | 0.958 |
| *city.MS:date* | 0.1259 | 0.115 | 0.276 | ***city.NM:HS*** | -0.7110 | 0.623 | 0.254 |
| *city.NE:date* | 0.1018 | 0.112 | 0.363 | ***city.OR:HS*** | 1.1928 | 0.912 | 0.191 |
| *city.NM:date* | 0.0545 | 0.117 | 0.642 | ***city.TX:HS*** | 0.0138 | 0.792 | 0.986 |
| *city.OR:date* | 0.1002 | 0.1114 | 0.368 | ***city.UT:HS*** | -0.9415 | 0.628 | 0.134 |
| *city.TX:date* | 0.2958 | 0.113 | *0.008* | ***city.WA:HS*** | 1.6107 | 0.819 | 0.049 |
| *city.UT:date* | 0.0940 | 0.1112 | 0.398 |  |  |  |  |
| *city.WA:date* | 0.0857 | 0.1124 | 0.446 |  |  |  |  |
