# Supplementary material for: Defining dual-axis landscape gradients of human influence for studying ecological processes
Source: PLoS One. 2021 Nov 18;16(11):e0252364. doi: 10.1371/journal.pone.0252364 (PMC8601559; doi:10.1371/journal.pone.0252364)
Supplement: Attachment — Submitted filename: PLOS_ONE_REVIEW.pdf [file pone.0252364.s003.pdf]

Review for:

Defining dual-axis landscape gradients of human influence for studying ecological processes

This paper describes a way to separate multiple axes of human modification on the landscape through the use of spatially weighted averaging of NLCD data via a Gaussian kernel and application of PCA, which is simple and provided intuitive results (a win win in my opinion). The statistical analysis as described appears sound, and using a common and easy to identify bird species helps alleviate any concerns I may have potentially had with using community science data from ebird (though you perhaps want to sell other readers on this in the methods section). I pretty much have one primary concern (top-level thought number 1), and then some relatively minor suggestions / improvements to the manuscript. I'm waiving anonymity (I'm Mason Fidino), so if there are any questions that arise from this review please reach out to me at mfidino@lpzoo.org.

Top-level thoughts

1. One aspect I've wondered about a lot, as someone who is also interested in how species distributions differ within and among cities, is how applicable PCA is when applied to multiple cities combined. Certainly, when applied this way PCA will identify the average gradient of urban intensification across cities, but then we do not know how well that average gradient applies to each city. This can also cause statistical issues in terms of model interpretation. For example, while the global mean of each gradient identified via PCA will be zero (assuming the data has been scaled), the city specific means when subset from that global gradient are not likely going to equal that global mean. Similarly, the standard deviation of each gradient will not be equivalent to the global standard deviation. Depending on the extent of these differences, I would imagine it could be very hard to compare effect sizes of regression coefficients from a global PCA that has then been subset among multiple cities (which is why we generally want to have mean = 0 and sd = 1 for a given continuous covariate).

There are, of course, other styles of PCA that help address this (e.g., multi-group PCA, or more recently network PCA), but it's something I've never really seen used in ecology (but perhaps should be when we start thinking about quantifying gradients across cities).

```
Eslami, A., Qannari, E. M., Kohler, A., & Bougeard, S. (2014). Multivariate analysis
of multiblock and multigroup data. Chemometrics and Intelligent Laboratory Systems,
133, 63-69.

Codesido, S., Hanafi, M., Gagnebin, Y., González-Ruiz, V., Rudaz, S., & Boccard, J.
(2020). Network principal component analysis: a versatile tool for the investigation
of multigroup and multiblock datasets. Bioinformatics.
```

The authors did apply single-city PCAs (available in supplemental material), but the explanation within the main text (and supplemental table) still have not especially assuaged the concerns I have here. For example, the single-city PCAs center on an individual city, whereas the multi-city PCA centers on the global average. How different the average of each variable is among cities is then could possibly have a

big influence on what is being observed. At a minimum, perhaps it would help to city-mean center your variables before applying the global PCA (and then not mean centering when calling `princomp()` or `prcomp()` in `R`)?

2. With this many cities, and given the interest in quantifying differences within and among cities, I wonder if it would help to treat city as a random effect within the model. Currently, all regression coefficients have to be interpreted relative to some baseline city for comparison whereas a multi-level model you can estimate an average response and deviation from that response for each city. Of course, this adds in the complexity of needing to do a Bayesian analysis, but even if the authors do not want to specify a model in JAGS, NIMBLE, or Stan the `ubms` package facilitates unmarked style analyses while adding in the ability to include `lme4` style random effects into the linear predictors for occupancy and detection. Definitely not necessary, but something to consider here.

https://kenkellner.com/ubms/index.html

3. Multi-city urban ecology research is still relatively new, and so there are not many papers out yet about how differences among cities may influence what we observe within a city. However, I'd encourage the authors to think about the results they found in the context of other multi-city studies (some that come to mind are below). Please note that while I try my best to not encourage authors to cite my papers in the review process, in Fidino et al. (2020) we specifically quantified how common urban mammals urbanization response differed across cities due to landscape level differences among cities, which I think is right in line what what you are observing with your own analysis.

```
Aronson, M. F., La Sorte, F. A., Nilon, C. H., Katti, M., Goddard, M. A., Lepczyk, C.
A., ... & Winter, M. (2014). A global analysis of the impacts of urbanization on bird
and plant diversity reveals key anthropogenic drivers. Proceedings of the royal
society B: biological sciences, 281(1780), 20133330.

Beninde, J., Feldmeier, S., Werner, M., Peroverde, D., Schulte, U., Hochkirch, A., &
Veith, M. (2016). Cityscape genetics: structural vs. functional connectivity of an
urban lizard population. Molecular ecology, 25(20), 4984-5000.

Fidino, M., Gallo, T., Lehrer, E. W., Murray, M. H., Kay, C., Sander, H. A., ... &
Magle, S. B. (2020). Landscape-scale differences among cities alter common species’
responses to urbanization. Ecological Applications, n/a (n/a), e2253. doi:
https://doi. org/10.1002/eap, 2253.

Gagné, S. A., Sherman, P. J., Singh, K. K., & Meentemeyer, R. K. (2016). The effect of
human population size on the breeding bird diversity of urban regions. Biodiversity
and conservation, 25(4), 653-671.
```

4. How universal these trends observed here are conditional on the cities used. As the paper only focuses on cities that range in populations from 200K to 500K I am still left wondering how applicable this is to smaller or larger cities. Certainly worth a few sentences to explore the idea in the discussion.

Introduction

Top-level thoughts

1. This manuscript gets right to the point, which is refreshing.

2. There is a little bit of passive voice that creeps in, especially with the use of 'understanding'. A small edit, but it would be nice to see these in the active voice and edit such sentences to remove some unnecessary gerunds.

3. As a near permanent change on the landscape, I often wonder why we equate urban intensity as a "disturbance." (Line 71). Would it be more clear to say that species richness is greatest at intermediate levels of urban intensification (or perhaps human modification to keep with the phrase you are using), when landscape heterogeneity is often at it's greatest?

4. The introduction uses the phrase "ecological processes" enough, which is perhaps a little too vague at times. For example, the manuscript states that fragmentation and human population density are not related to a range of ecological processes (line 75), but it would help to be more specific about what these ecological processes are.

Line by line comments

Line 41: I understand the point of this sentence, but the wording makes it difficult. I think it is because it is unclear what 'expands' is acting upon, the link between 'human population' and 'ecological footprint', and the assumption that this means natural landscapes are being transformed due to this.

Line 72: replace "are more variable," with "vary"

Line 75: missing a word here.

Methods:

Top-level thoughts

1. A sentence in the PCA section of the methods wherein you give a heads up that you'll describe these gradients within the results section would be helpful. I got a little confused at what is only presented in the methods, and it had me wondering whether or not the manuscript was actually going to define the primary gradients captured in the analysis, provide the loadings, etc (which was in the results).
2. A little more explanation about what a "location" is could be helpful. Were surveys thinned so you could assume they were spatially independent?

Line by line comments

Line 119: What do these percentages represent? Composition across all landscapes? If so, that does not indicate that landscape composition is similar. We would instead be interested in how much these dominant landscape types vary among cities. For example, based off of table 1, forest landcover is clearly not similar across cities.

Line 173: At a minimum cite either the MacKenzie et al. book, a paper, etc. for occupancy modeling.

Results

Top-level thoughts

1. Loadings are just as important as the description of what the gradient represents. I'm assuming that is what is listed in Table 3 (PC1, PC2, and PC3), but a little more explanation in the table header can make that explicit.

2. What are the values presented in parentheses? Regression coefficients and p values? Regression coefficients and standard errors? Please provide a little more information.

3. Figure 4 does not especially capture the variation that is being explained. Something similar to Fig 3 for each city could help with this (perhaps replacing figure 3 if there are space constraints, which is focused on detection with one that is focused on within vs among city variability in occupancy).

Discussion

Top-level thoughts

Since there was no comparison here to one-dimensional approaches for urban gradients I am not certain how the analysis described here represents a challenge. No doubt there is still merit in the PCA approach used here, but perhaps it would be better to sell the reader on the insights which you may have missed otherwise.

Thinking about it even further, and perhaps I'm getting a little off-topic, but urban ecologists have tried to capture urban intensity in a variety of ways. Traditionally, we used categorical metrics (e.g., urban vs rural) which are not useful for a variety or reasons. Following this we tried more continuous metrics that were simple to calculate like distance to city center, which while better than categories it effectively assumes a city is an onion, steadily decreasing in urbanization from it's core through each "onion layer". Modern cities, however, are not onions, so again not a great approach. Currently, I would argue we are in the "one-dimensional" vs "PCA" style approach like this manuscript details. While I fit into the PCA camp a bit more, I'm still a little agnostic to which approach is better, because both have their uses. For example, I would argue that a one-dimensional approach makes it easier to fit multi-level models because that single gradient can provide you a way to incorporate within vs among city variation as two seperate continuous covariates (e.g., average of a given gradient within each city, and then a city-mean centered site-specific gradient, Fidino et al. 2020).

Figures.

Figure 3 legend says it's about detection but the y-axis says occupancy.
