# Supplementary material for: Defining dual-axis landscape gradients of human influence for studying ecological processes
Source: PLoS One. 2021 Nov 18;16(11):e0252364. doi: 10.1371/journal.pone.0252364 (PMC8601559; doi:10.1371/journal.pone.0252364)
Supplement: Attachment — Submitted filename: Response to Reviewers.docx [file pone.0252364.s004.docx]

Response to Reviewers

Reviewer One:

This paper describes a way to separate multiple axes of human modification on the landscape through the use of spatially weighted averaging of NLCD data via a Gaussian kernel and application of PCA, which is simple and provided intuitive results (a win win in my opinion). The statistical analysis as described appears sound, and using a common and easy to identify bird species helps alleviate any concerns I may have potentially had with using community science data from ebird (though you perhaps want to sell other readers on this in the methods section). I pretty much have one primary concern (top-level thought number 1), and then some relatively minor suggestions / improvements to the manuscript. I'm waiving anonymity (I'm Mason Fidino), so if there are any questions that arise from this review please reach out to me at [mfidino@lpzoo.org](mailto:mfidino@lpzoo.org).

- Hello Mason, we thank you very much for your thorough and generally positive review, and we would like to extend an extra thank you for being candid and offering to supply additional insight if necessary. Ultimately, the review you provided was very clear and offered concrete guidance on how to address the issues raised, therefore we did not find it necessary to contact you directly.

**Top-level thoughts**

1. One aspect I've wondered about a lot, as someone who is also interested in how species distributions differ within and among cities, is how applicable PCA is when applied to multiple cities combined. Certainly, when applied this way PCA will identify the average gradient of urban intensification across cities, but then we do not know how well that average gradient applies to each city. This can also cause statistical issues in terms of model interpretation. For example, while the global mean of each gradient identified via PCA will be zero (assuming the data has been scaled), the city specific means when subset from that global gradient are not likely going to equal that global mean. Similarly, the standard deviation of each gradient will not be equivalent to the global standard deviation. Depending on the extent of these differences, I would imagine it could be very hard to compare effect sizes of regression coefficients from a global PCA that has then been subset among multiple cities (which is why we generally want to have mean = 0 and sd = 1 for a given continuous covariate).

There are, of course, other styles of PCA that help address this (e.g., multi-group

PCA, or more recently network PCA), but it's something I've never really seen used in

ecology (but perhaps should be when we start thinking about quantifying gradients

across cities).

Eslami, A., Qannari, E. M., Kohler, A., & Bougeard, S. (2014). Multivariate analysis

of multiblock and multigroup data. Chemometrics and Intelligent Laboratory Systems,

133, 63-69.

Codesido, S., Hanafi, M., Gagnebin, Y., González-Ruiz, V., Rudaz, S., & Boccard, J.

(2020). Network principal component analysis: a versatile tool for the investigation

of multigroup and multiblock datasets. Bioinformatics.

The authors did apply single-city PCAs (available in supplemental material), but the explanation within the main text (and supplemental table) still have not especially assuaged the concerns I have here. For example, the single-city PCAs center on an individual city, whereas the multi-city PCA centers on the global average. How different the average of each variable is among cities is then could possibly have a big influence on what is being observed. At a minimum, perhaps it would help to citymean center your variables before applying the global PCA (and then not mean centering when calling princomp() or prcomp() in R)?

- Thank you for bringing these approaches to our attention, they certainly do seem to be well-suited for examining variation within- and among- regions in a multi-region gradient analysis. Further, your point is well taken, with an exploratory analysis such as PCA applied jointly to all cities makes it difficult to understand how well the combined principal component applies to each city. This is why we also conducted city-specific PCA, which showed regional variation in variable loadings, but consistency in the interpretation of dominant principal components. Your suggestion, to city-mean center the data prior to PCA essentially represents a middle ground between our city-specific analysis and global PCA. Both analyses resulted in two dominant components that explained spatial heterogeneity in the built (hard) and agricultural (brown) aspects of human dominated landscapes. Because this work ultimately serves to provide a proof of concept for multi-dimensional landscape gradients, we believe the inclusion of city-specific and combined analyses adequately demonstrate the reliability of landscape scale PCA in identifying hard-soft and brown-green landscape gradients.

2. With this many cities, and given the interest in quantifying differences within and among cities, I wonder if it would help to treat city as a random effect within the model. Currently, all regression coefficients have to be interpreted relative to some baseline city for comparison whereas a multi-level model you can estimate an average response and deviation from that response for each city. Of course, this adds in the complexity of needing to do a Bayesian analysis, but even if the authors do not want to specify a model in JAGS, NIMBLE, or Stan the ubms package facilitates unmarked style analyses while adding in the ability to include lme4 style random effects into the linear predictors for occupancy and detection. Definitely not necessary, but something to consider here. <https://kenkellner.com/ubms/index.html>

- A mixed effects structure with city-specific random effects would certainly have been appropriate for this analysis, and indeed the ubms package looks like an excellent contribution that we are looking forward to exploring in more detail. We agree that as we look to extend this analytical approach beyond the proof-of-concept phase, i.e., when our scope of inference shifts to inferring national patterns using a subset of cities as samples, such a random effects structure will be a sensible option.

3. Multi-city urban ecology research is still relatively new, and so there are not many papers out yet about how differences among cities may influence what we observe within a city. However, I'd encourage the authors to think about the results they found in the context of other multi-city studies (some that come to mind are below). Please note that while I try my best to not encourage

authors to cite my papers in the review process, in Fidino et al. (2020) we specifically quantified how common urban mammals urbanization response differed across cities due to landscape level differences among cities, which I think is right in line what what you are observing with your own analysis.

Aronson, M. F., La Sorte, F. A., Nilon, C. H., Katti, M., Goddard, M. A., Lepczyk, C.

A., ... & Winter, M. (2014). A global analysis of the impacts of urbanization on bird

and plant diversity reveals key anthropogenic drivers. Proceedings of the royal

society B: biological sciences, 281(1780), 20133330.

Beninde, J., Feldmeier, S., Werner, M., Peroverde, D., Schulte, U., Hochkirch, A., &

Veith, M. (2016). Cityscape genetics: structural vs. functional connectivity of an

urban lizard population. Molecular ecology, 25(20), 4984-5000.

Fidino, M., Gallo, T., Lehrer, E. W., Murray, M. H., Kay, C., Sander, H. A., ... &

Magle, S. B. (2020). Landscape-scale differences among cities alter common species’

responses to urbanization. Ecological Applications, n/a (n/a), e2253. doi:

https://doi. org/10.1002/eap, 2253.

Gagné, S. A., Sherman, P. J., Singh, K. K., & Meentemeyer, R. K. (2016). The effect of

human population size on the breeding bird diversity of urban regions. Biodiversity

and conservation, 25(4), 653-671.

- We agree that placing our work into the broader context of multi-city urban ecology is very important and we thank you for pointing us to these references! We have now added relevant context and citations in discussion in lines 458-468 as follows:

“Multi-city analysis of urban ecosystems has experienced rich growth in recent years. This work has highlighted the negative and positive potential impacts of urbanization on biodiversity, while stressing the importance of the regional landscape context in driving the direction and magnitude of biological response [80–82]. Still, multi-region analysis remains hampered by inconsistencies in study design and methodological limitations [83,84]. The multi-dimensional, dual-axis understanding of spatial heterogeneity we describe has the potential to improve and standardize existing approaches to producing ecologically relevant landscape metrics leading to improvements in multi-region research and can provide valuable and generalizable insight into patterns of ecological response in within and across human-dominated systems.”

4. How universal these trends observed here are conditional on the cities used. As the paper only focuses on cities that range in populations from 200K to 500K I am still left wondering how applicable this is to smaller or larger cities. Certainly worth a few sentences to explore the idea in the discussion.

- This is certainly true, and thanks for pointing this out. While we believe the specifics of robin response may have differed in larger (or smaller) urban regions, the central take home message of our research would have remained. That is, the importance of considering multiple axes of variation in landscape gradients, and regional variation in biotic response. We have added some relevant discussion in lines 424-432:

“City-specific variation in robin response landscape heterogeneity reiterates the importance of landscape context on biotic response. The size (i.e., spatial extent) and density (i.e., human population) of human-dominated landscapes significantly impacts the direction and magnitude of biotic response, with larger and more densely populated cities typically resulting in a stronger negative response [78,79]. Had our analysis centered on larger or smaller urban regions the specifics of robin response may have differed, however, our core findings – the importance of considering multiple landscape gradients and regional variation in response – would likely have remained. Though, additional research into the impacts of size of human-dominated landscapes on the use of multi-dimensional landscape gradients is warranted.”

**Introduction**

**Top-level thoughts**

1. This manuscript gets right to the point, which is refreshing.

- Thank you!

2. There is a little bit of passive voice that creeps in, especially with the use of 'understanding'. A small edit, but it would be nice to see these in the active voice and edit such sentences to remove some unnecessary gerunds.

- Thanks for pointing this out, we have done our best to maintain active voice throughout the manuscript

3. As a near permanent change on the landscape, I often wonder why we equate urban intensity as a "disturbance." (Line 71). Would it be more clear to say that species richness is greatest at intermediate levels of urban intensification (or perhaps human modification to keep with the phrase you are using), when landscape heterogeneity is often at it's greatest?

- That is an excellent point, and we have edited the text accordingly. However, even when considering “disturbance” in the ecological sense, ecosystems often do not rebound to the same pre-disturbance state. So, in that case urbanization can be thought of as a significant disturbance where the resulting ecological state is a new ecosystem type (eg urban). Perhaps it is akin to a disturbance such as large fires that may push forested savannahs to become grasslands. This (below) is an interesting read on the concept of “disturbance” in urban systems
- Grimm NB, Pickett STA, Hale RL, Cadenasso ML (2017) Does the ecological concept of disturbance have utility in urban social-ecological-technological systems? Ecosyst Heal Sustain 3:e01255. https://doi.org/10.1002/ehs2.1255

4. The introduction uses the phrase "ecological processes" enough, which is perhaps a little too vague at times. For example, the manuscript states that fragmentation and human population density are not related to a range of ecological processes (line 75), but it would help to be more specific about what these ecological processes are.

- We understand that “ecological processes” is somewhat vague, our intent was to avoid unnecessary detail and specificity, but we recognize that some is needed. We have added a few examples in line 78 as follows:

“a range of ecological processes (e.g., population size, species distribution)”

**Line by line comments**

Line 41: I understand the point of this sentence, but the wording makes it difficult. I think it is because it is unclear what 'expands' is acting upon, the link between 'human population' and 'ecological footprint', and the assumption that this means natural landscapes are being transformed due to this.

- We tried to improve the wording for this sentence. It has been changed to:

“Rapid expansion of the global human population has led to increasing concern for natural systems and biodiversity.”

Line 72: replace "are more variable," with "vary"

- Replaced

Line 75: missing a word here.

- Thank you. Actually, this was simply a case of poor sentence construction. We have tried to re word it for clarity

**Methods:**

**Top-level thoughts**

1. A sentence in the PCA section of the methods wherein you give a heads up that you'll describe these gradients within the results section would be helpful. I got a little confused at what is only presented in the methods, and it had me wondering whether or not the manuscript was actually going to define the primary gradients captured in the analysis, provide the loadings, etc (which was in the results).

- We added the following in line 176:

“Output for our PCA analyses are reported in the Results section under *Landscape Gradient Analysis*.”

2. A little more explanation about what a "location" is could be helpful. Were surveys thinned so you could assume they were spatially independent?

- “location” here refers to each unique eBird location (hotspot) used in analysis. We thinned the data not in order to achieve spatial independence, but to reduce the discrepancies in sample size between the ten study cities. We tried to make this clearer by adding additional context in line 194 (relevant changes are in bold here):

“Because there was substantial variation in the number of eBird locations in each city (i.e., each unique fixed point eBird survey site), and to improve balance and reduce regional bias in sample size, the data were randomly thinned to a maximum of 250 locations.”

**Line by line comments**

Line 119: What do these percentages represent? Composition across all landscapes? If so, that does not indicate that landscape composition is similar. We would instead be interested in how much these dominant landscape types vary among cities. For example, based off of table 1, forest landcover is clearly not similar across cities.

- These values represent the aggregate composition of these dominant land cover categories in all cities considered jointly. Thank you for pointing out that this was unclear. We have tried to make this clearer by changing the text to:

“Landscape composition was fairly evenly split between three dominant lands cover categories when aggregated among all cities: developed (20.03%), forests (23.98%), and agriculture (31.28%) and contained fifteen of the nineteen Anderson Land-Cover classes used by the NLCD. The remaining four (‘Perennial Ice-Snow’, ‘Dwarf Scrub’, ‘Sedge-Herbaceous’, ‘Lichen’) are restricted to Alaska or high elevation locations.”

Line 173: At a minimum cite either the MacKenzie et al. book, a paper, etc. for

occupancy modeling.

- We cited the Mackenzie et al (2002) *Ecology* paper in line 186

Mackenzie DI, Nichols JD, Lachman GB, et al (2002) Estimating Site Occupancy Rates When Detection Probabilities Are Less Than One. Ecology 83:2248–2255

**Results**

**Top-level thoughts**

1. Loadings are just as important as the description of what the gradient represents. I'm assuming that is what is listed in Table 3 (PC1, PC2, and PC3), but a little more explanation in the table header can make that explicit.

- We have updated to caption for Table 3 to more clearly describe all aspects of the table. It now reads as follows:

“Dominant Principal Component axes. The Obs. Freq. column displays the percent composition of each land cover category in all cities combined. For each principal component, standard deviation, percent variance explained, and rotated variable loadings are displayed. Variables with a strong weight are in bold. The first two axes were selected because cumulative variance exceeded the 22.1% broken stick threshold.”

2. What are the values presented in parentheses? Regression coefficients and p values? Regression coefficients and standard errors? Please provide a little more information.

- We have updated all commas here with a plus-minus sign (±). Values in parentheses represent (estimate± SE). Line 312, where reporting model estimates begins, currently reads as follows:

“There was a significant quadratic effect (*estimate± SE)* of survey date on detectability (-0.012± 0.002), such that detection probability increased, reached a peak, and declined”

3. Figure 4 does not especially capture the variation that is being explained. Something similar to Fig 3 for each city could help with this (perhaps replacing figure 3 if there are space constraints, which is focused on detection with one that is focused on within vs among city variability in occupancy).

- Thank you for this suggestion. Our goal with Figure 4 is to visualize the variation in occupancy along both landscape gradients simultaneously rather than the marginal response along one gradient as in Figure 3. Our preference is to keep the figures as is as we feel it best represents robin occupancy along both hard-soft and brown-green gradients. If this is a major concern, we are happy to reconsider this position.

**Discussion**

**Top-level thoughts**

Since there was no comparison here to one-dimensional approaches for urban gradients I am not certain how the analysis described here represents a challenge. No doubt there is still merit in the PCA approach used here, but perhaps it would be better to sell the reader on the insights which you may have missed otherwise.

Thinking about it even further, and perhaps I'm getting a little off-topic, but urban ecologists have tried to capture urban intensity in a variety of ways. Traditionally, we used categorical metrics (e.g., urban vs rural) which are not useful for a variety or reasons. Following this we tried more continuous metrics that were simple to calculate like distance to city center, which while better than categories it effectively assumes a city is an onion, steadily decreasing in urbanization from it's core through each "onion layer". Modern cities, however, are not onions, so again not a great approach. Currently, I would argue we are in the "one-dimensional" vs "PCA" style approach like this manuscript details. While I fit into the PCA camp a bit more, I'm still a little agnostic to which approach is better, because both have their uses. For example, I would argue that a one-dimensional approach makes it easier to fit multi-level models because that single gradient can provide you a way to incorporate within vs among city variation as two seperate continuous covariates (e.g., average of a given gradient within each city, and then a city-mean centered site-specific gradient, Fidino et al. 2020).

- As you have noted, lack of direct comparison to a pure one-dimensional or categorical approach here limits our ability to directly describe improvements levied by multi-dimension axes. However, use of a single dominant principal component in ecological analysis is common, as you have noted. In our analysis of robin occupancy we compared single-axis and dual-axis and found overwhelming support for dual-axis measures. In our discussion we noted the importance of relying on dual-axis measures as follows:

“We saw that robin occupancy was demonstrably influenced by both axes of human-modification across the continental United States, suggesting that a continued reliance on one-dimensional landscape descriptors may result in ecosystem pattern being misinterpreted as inherent stochasticity (e.g., noise), when in fact it reflects an overlooked component of the landscape. Specifically in our context, an analysis using a conventional hard-soft gradient would have overlooked the value of green (natural) landscapes integrated in hard (urban) regions for robin (Fig. 4).”

**Figures**.

Figure 3 legend says it's about detection but the y-axis says occupancy.

- Thank you for noticing this, we have corrected our error.

Reviewer #2:

I appreciate the efforts in suggesting a standardized approach for defining multi-dimensional gradients, as this is key for understanding species responses to human modifications (as shown for the American Robin) and for informing management and conservation efforts.

- Thank you very much for the positive assessment of our work, and for your detailed and thorough review of our manuscript. Your comments, critiques and suggestions were very helpful in refining and improving the clarity and quality of this manuscript.

However, previous attempts in this direction have been made and have already suggested a multi-dimensional approach instead of using one-dimensional measure (e.g. Luck and Wu 2002; Hahs and McDonnell 2006; Schwarz 2010; Modica et al. 2012; Suarez-Rubio and Krenn 2018), thus this should be acknowledged and discuss in the manuscript.

- Thank you for highlighting this. Yes, there have been numerous notable attempts at standardizing urbanization gradients in the literature, with some advocating for multiple metrics over a single measure. Our work relies on an approach that differs from prior attempts, including those you’ve mentioned here, in a few key respects. First, the majority of multi-metric approaches have used multiple *univariate* metrics to represent urbanization in their work, and with the exception of Hahs and McDonnell 2006 (see figure 7 there), the multiple univariate metrics are related to urbanization individually. We, in contrast, compressed our data into multivariate metrics of landcover change and apply them jointly to ecological processes (as suggested by Berland and Manson). Finally, our work differs in that we examine landscape change along multiple axes of human-mediated landscape change, not solely urbanization as has typically been the case. This is a critical distinction that extends our work beyond the urban centers analyzed elsewhere, to incorporate the broader urban-residential-agro system.

We have added a brief discussion of the multi-measure literature and how our work is distinct in lines 380-391 as follows: “Multiple metrics have been used to analyze and quantify spatial change in human-dominated systems. Large suites of input variables ranging from landscape configuration measures to human population density have been used to identify multiple important features of change in urban landscapes in several notable instances [9,65,67,68]. In most of these cases, however, multiple univariate measures are identified (e.g., using multi-variate analyses) as representative of landscape change along urban-rural gradients, while Berland and Mason [67], noted that dominant factors or principal components could perhaps be used to directly represent urbanization rather than selecting the variables with highest loading. Ultimately, regardless of the number of metrics utilized, or how they were derived, prior multi-metric research has tended to focus on identifying one aspect of landscape change, namely urbanization. Furthermore, despite identifying multiple important measures, none of these explicitly promote a dual-axis or multi-dimensional application of these measures.”

Although most statistical analyses were appropriate, a Factor Analysis (FA) should be performed instead of the PCA. du Toit and Cilliers (2011) carefully argue why PCA is not the correct method when the purpose is identifying uncorrelated axes of variation and distinguishing the different aspects forming a multi-dimensional urbanization gradient ‒despite its earlier use. Additionally, subsequent studies used this methodology in their research e.g. Berland and Manson (2013), Leveau (2013), Suarez-Rubio and Krenn (2018). The manuscript's main conclusions are based on the PCA results, which might change considerably if the data were reanalyzed using FA.

- Thank you for bringing up du Toit and Cillier’s (2011) comparison of FA and PCA. Their work does an excellent job of identifying the conceptual and statistical differences between FA and PCA, and lends support for FA for their specific application. As the authors note, FA identifies factors based on shared variance in the input variables such that factors represent covariance in the inputs and assumes the existence of a latent variable embedded in the reduction process. Furthermore, rotating factors in FA allows for more intuitive interpretation of results. In contrast, PCA considers variance shared among variables and variance explained by individual variables such that dominant components explain the maximum amount of variance in the input variables. They conclude that “for the purpose of identifying uncorrelated representative measures in the current study, the FA method is much more suitable”. Finally, du Toit and Cillier used FA as a method to identify a subset of univariate input variables for use in subsequent analysis.

In contrast to du Toit and Cillier’s use of FA for variable selection, our objectives were identify gradients of landscape variation in human dominated landscapes and produce continuous landscape metrics in these regions, not to identify and select individual variables correlated with change in these landscapes. We recognize that FA has been used to produce continuous metrics based on factor scores, however, in the many cases, the variables included in FA included biologic information, landcover data, and human socio-economic and demographic data. Because FA identifies factors based on shared variance among variables, it is particularly well suited to identifying landscape gradients in these sorts of data sets (e.g., a factor describing “urbanization” strongly loaded for impervious surface and human population density). In contrast, we opted for a more streamlined approach that considered only landcover data in our PCA (see below for rationale in selection of spatial data). PCA considers both individual variance and shared variance when estimating loading scores, which we believe makes it better suited for identifying gradients of variation in landcover data where one or more variable may weigh strongly on a PC loading without sharing significant variance. For these reasons, we maintain that PCA was the best approach to analyzing landcover data and identifying significant patterns of variation in the data. We have added clarification in lines 169-172:

“PCA one of several methods for summarizing a large number of potentially correlated variables into fewer uncorrelated axes of variation (others include factor analysis, non-metric multidimensional scaling, correspondence analysis), and it is particularly well suited to exploratory ordination and gradient analysis [50].”

As well as an important note in the discussion reinforcing the fact that our work aims to highlight the value of considering multiple axes of variation, not to prioritize the use of PCA over any other multi-variate statistical approach. Lines 386-394:

“In addition to PCA, other multi-variate methods have been suggested as alternatives when identifying landscape gradients, such as factor analysis [9], and non-metric multidimensional scaling [68]. More recently, PCA approaches better account for similarities and differences in multi-block data have been developed [69], which may be particularly applicable when analyzing landscape gradients in multiple regions simultaneously. Our analysis has demonstrated the importance of considering multiple axes of variation in landscape gradients, and fits within a methodological framework centered on transparent and ecologically informed analysis [3]. Ultimately, the multi-variate method selected by researchers should be informed by the study’s goals, objectives, and types of data available [50].”

A better description of the methods, in particular the landscape quantification is needed. Although it was mentioned that the landscape analysis followed the framework of a previous publication, it is important for the repeatability of the study to briefly describe how the quantification was performed. In Table 2, a description of decisions is included but it is unclear how the physical land-cover differ from the NLCD and from where the land-use data are coming from. In addition, it seems that the study only included landscape composition variables, why configuration variables are not included, this was shown to be relevant in previous studies (see references in paragraph above).

- We recognize that multiple landscape configuration and human-demographic metrics are often incorporated into landscape gradients in urban systems. These additional data offer an additional lens on the structural and human-dimensions of landscape change, but typically require additional analysis to produce (i.e., configuration metrics) or are difficult to access in some areas (i.e., human socio-demographic data). Our research objectives were to produce landscape gradients for use in ecological analysis in a streamlined manner that is reproducible across scales and in different regions. Therefore, we chose to include only landcover data, which is freely available, accurate, and has nearly global coverage. We believe that landscape configuration or demographic data are extremely valuable, and should be considered in addition to the landscape gradients described depending on a given research question.

Minor comments:

Line 28 It would be nice if the name of the focal species is mentioned in the abstract.

- We have added the name of the focal species to the abstract as requested.

Line 102 As “medium size city” have different meanings in other urban settings around the world, it will be relevant to include the average size of the cities studied and in Table 1 include the size of each city.

- We acknowledge that what is considered a “medium-sized city” varies in different regions and contexts. For this reason, we specified the population range of cities included in our study to provide a context for size. Because of variation in exurban sprawl and size of official city boundaries, we considered population size to be a reliable measure of approximate city size, with larger population cities having denser urban cores and more extensive exurban sprawl. Population size is included in Table 1 to provide context for readers.

Line 220 There are several tables included in S1, to which one in particular this refers to?

- Thank you for pointing this out, we have added the table number as follows (S1, Table S1.1)

Line 287 Table 3 or 4?

- This was a mistake and should reference Table 4 as you have noted. We have made the appropriate change.

Line 304-306 Where are these results depicted?

- The detection probability results are depicted in Fig. 3 and in Supplement S2. We had referenced these at the start of the paragraph, but have moved the figure reference to the end where it is more appropriate.

Lines 320-326 I assume that these results are presented in S2 Table 3, if so, please make sure that the numbers are the same as some of them are not (e.g. -1.68 vs. -1.72).

- Thank you for catching these mistakes, we have corrected them in the main text.

Line 342 I do not think this is supported by Fig. 3.

- Reference to Fig. 3 has been updated to Fig.2

Line 369 Although the GlobCover data is a good source, it is quite old (2009), so better also to include the Copernicus Global Land Cover.

*And*

Lines 351-361 Please refer to the comment above.

- Thank you for the suggestion, we have included the more recent Copernicus Global Landcover as well

Line 383 to which species this refers to, the American Robin?

- The species referred to here is general, in other words, in any land sharing or lands paring effort, the winners and losers in the ecosystem will depend on the landscape context of the study system. We have tried to make this more clear as follows:

“Furthermore, the species that will benefit or suffer most from any specific sharing or sparing management, depends entirely on the landscape context within which they are evaluated.”

Table 1: There is no need to include % next to each number under the land-cover classes.

- Thank you, we understand that there is no need to keep the % signs here, therefore we have removed them from the table.

Fig.1 For the international readers not familiar with the location of the cities in the US, including the name of the city in the map or even a number as an ID of the city will be highly appreciated. In addition, it will be better to be consistent in the way you refer to them, sometimes the name of the city is used (Line 78) and sometimes the state where the city is located (Line 318, S2 Table 3).

- This is a good suggestion, thank you. We have added city names where the state names are used (eg line 318 and S2). We have also added numbers to figure 1 with city names in the figure caption.
- Fig. 2 In the lower left corner, I think that % of forest should be high and not low.Thank you for catching this error, we have corrected the figure accordingly.

Fig. 3 Based on the text (Lines 302-304), the y-axis in the figures should be detection probability, correct?

- The figure depicts detection probability and has been corrected accordingly.

S1 will benefit from including first the information about the spatial extent (city window) and the then the smoothing scale and city specific analysis.

- Excellent suggestion regarding the ordering of S1, we have changed the organization to have city window included first. Thank you

The manuscript is well-written. But there are few things that should be revised:

Line 233 Replace boldened by bold.

- Done, we have changed ‘boldened’ to ‘bold’ as suggested

Line 236 It should be 11.1%

- We have corrected the error replacing 11.2% with 11.1%

Line 132 and 400 Instead of including the citation in author-year format include the corresponding number.

- Thank you for catching these errors, we have resolved them.

References should be checked for accuracy and consistency, e.g. 6, 12, 22, 35, 37, 69.

- We have checked references, and they all now appear to be accurate and consistent

References:

Berland A, Manson SM (2013) Patterns in Residential Urban Forest Structure Along a Synthetic Urbanization Gradient. Annals of the Association of American Geographers 103:749-763

du Toit MJ, Cilliers SS (2011) Aspects influencing the selection of representative urbanization measures to quantify urban-rural gradients. Landscape Ecol 26:169-181

Hahs AK, McDonnell MJ (2006) Selecting independent measures to quantify Melbourne's urban–rural gradient. Landsc Urban Plan 78:435-448

Leveau LM (2013) Bird traits in urban-rural gradients: how many functional groups are there? J Ornith 154:655-662

Luck M, Wu J (2002) A gradient analysis of urban landscape pattern: a case study from the Phoenix metropolitan region, Arizona, USA. Landscape Ecol 17:327-339

Modica G, Vizzari M, Pollino M, Fichera CR, Zoccali P, Di Fazio S (2012) Spatio-temporal analysis of the urban-rural gradient structure: an application in a Mediterranean mountainous landscape (Serra San Bruno, Italy). Earth Syst Dynam 3:263-279

Schwarz N (2010) Urban form revisited—Selecting indicators for characterising European cities. Landsc Urban Plan 96:29-47

Suarez-Rubio M, Krenn R (2018) Quantitative analysis of urbanization gradients: a comparative case study of two European cities. Journal of Urban Ecology 4:1-14
