# Supplementary material for: Defining dual-axis landscape gradients of human influence for studying ecological processes
Source: PLoS One. 2021 Nov 18;16(11):e0252364. doi: 10.1371/journal.pone.0252364 (PMC8601559; doi:10.1371/journal.pone.0252364)
Supplement: Attachment — Submitted filename: Response_to_Reviewers_pone.docx [file pone.0252364.s005.docx]

We would once again like to extend our deepest thanks to the excellent reviews by Mason Fidino and the anonymous reviewer. Their comments, edits, and suggestions have dramatically improved the quality of our manuscript, thank you!

Tables 1 & 4.  Right justify all numbers.

Numbers are right justified

Table 1.  Remove % signs within body of table.  Add a statement to the caption that numbers are percent cover, or some such.

% signs have been removed, and relevant statement added to caption.

Table 3.  Why isn’t font size of numbers consistent throughout?  Why are some numbers italicized?

Thank you, numbers have been resized and italics removed

Do a ‘Find’ for all uses of ‘which’.  Ensure that the word preceding which is followed by a comma, e.g. Line 88, a comma should follow ‘approach’. I noticed at least 3 locations were the comma is missing.

Thank you for noticing this grammatical error. Corrected

Consolidate all information on your focal species and why this particular species into one spot.  Currently, the information is spread out in three spots and is redundant (lines 95-96, 161-162, 188-191).  Also, if you decide you want to refer to the scientific name more than once, then the standard practice is to spell the name of the genus out fully on first use and thereafter abbreviate to just the first letter, i.e. *T. migratorius*.

We appreciate this suggestion, however, after reviewing the manuscript, we believe that it is best organized as is. The majority of the important life history information on the study species is located in lines 188-191. The information in lines 95 and 96 is a brief reference to the study species in a summary of the manuscript’s objectives, while the reference in lines 161-162 is important information for the statistical methodology described in that section.

We have changed the second reference to the genus species name to read as *T. migratorious*.

Line 82. Replace ‘despite the fact that’ with ‘even though’.

Changed

Line 157. ‘usinga’ should be ‘using a’.

Revised

Line 168-180. Use the same font & size for PCA throughout. My preference is for it to be the same as the general text.

All occurrences of PCA have been changed to match the style of the general text.

Line 168. Should be ‘PCA is’.

Revised

Line 170. Delete the extra space before ‘and’.

Revised

Line 171. Add a space after the period.

Revised

Line 298. Missing ‘to’.

‘To’ has been added, thank you

Line 300. Add a comma after ‘date’.

Revised

Line 358. Delete second comma.

Revised

Line 366. Should be ‘the human footprint’.

Revised

Line 374. Should be ‘the landscape’.

Revised

Lines 381-385. Might be better to split into two sentences for clarity.

Revised

Line 401. Should be ‘approaches that’.

Revised

Line 407. Add a space after the period.

Revised

Lines 415-416. ‘Viewing’ and ‘views’?  Maybe reword?

This text has been reworded for clarity

Line 429. ‘suggest’ should be ‘suggests’.

Revised

Lines 432-436. Clarity lacking.  Needs modified.  Also, likely better to split into two sentences.

The text has been split into two sentences to improve clarity and readability

Line 433. ‘species have investigated’??  The species investigated?  I don’t think so. – This has been changed to “research on the species where single landscape gradients were considered in isolation”

Revised

Line 437. Missing ‘to’.

Revised

Line 451. Should be ‘robins’.

Revised

The revisions the authors made are fantastic. I think the only thing that really needs to be added in the discussion is a bit on how interpretation of city-specific regression coefficients from the global PCA approach. Basically, acknowledge that city-specific means of the PCA may vary from the global mean, and how that can influence interpretation of effect size. This isn't a problem with this approach, but I would hate for someone to get excited by this technique, use it, and then compare effect size among cities via the magnitude of regression coefficients. By giving a reader a roadmap to model interpretation with your approach, you'll hopefully ensure that it gets used effectively and correctly.

We have added the following text to lines 410-412 “It is important to note, however, that city-specific means in our global analysis which may influence interpretation and comparison of effect size between groups.”


Small line by line comments:

Line 179: Provide some justification to the reader about why you also conducted this analysis separately (e.g,. determine how well the combined (i.e., all cities) gradient described city-specific gradients).

Table 1: Shouldn't the numeric data be right justified? 🡪 **DONE**

Line 296: You report occupancy probabilities later as values between 0 and 1 instead of a percentage I'd be consistent in the way you report it. My personal preference is [0-1], but that is what it is, a preference. 🡪 **we have changed these to 0-1 scale**

Line 403: I know what you mean here, but readers unfamiliar with the term 'multi-block' coul be confused. Also, in this case, I think you actually mean 'multi-group'. I believe that multi-block is grouping among covariates (column wise) while multi-group means there is categorical structure of the same covariates (row wise). Maybe modify to "...differences in multi-group (e.g., multi-city) data..." 🡪 **Thanks for suggestion, this has been added**

Figure 3: Remove the underscores in Salt_Lake_City, and perhaps add the state abbreviations (e.g., because there is a Portland, Maine as well). 🡪 **DONE**

Cheers,
Mason Fidino

Reviewer #2: Thank you for addressing my previous comments. The clarity of the manuscript has improved and key points were acknowledged and added. However, there are minor things, mostly related to style, that should be considered. This is particularly important as PLOS ONE does not copyedit accepted manuscripts.

L82 Replace ‘despite the fact that’ by ‘although’

Revised

L113, 289 Please make sure that the heading included here match the ones included in the Appendix.

Revised

Table 1 This was previously mentioned, but the changes were not made. The percentages in each of the columns are not needed, please remove them.

Revised

L137-143/Table 2 The description of the methodology included in this paragraph is not clear. I had trouble following the procedure and although you cited Table 2, that did not help. What is the difference between ‘land-cover categories’ mentioned in ‘landscape features’ and the ‘land-cover data’ mentioned for the spatial data? You cited a previous publication, but to allow repeatability of the study it is important to clearly describe these steps.

We have done our best to improve this portion of the methods, and have revised it as follows “Our decisions regarding the types of landscape features relevant for analysis, the data to represent those features, and the spatial scales of analysis were made to reflect a typical ecological analysis - definitions of, and justification for, these decisions are provided in Table 2”

L286 It should be ‘x’

Revised

L354 Remove ‘configuration’. The study does not quantify the configuration of the cities, so including here the word ‘configuration’ is misleading. The information included in Table 1 in S1 Appendix includes only the proportion of land-cover i.e., composition.

Revised

L415-417, L437-438 Please revise the grammar of these sentences.

Revised

References There are many mistakes in the list of references. I mentioned here only few examples. In some cases each word of the title is capitalized in other instances it is not. In 6, Beggs J. editor, should be removed. In 22 first names should be replaced by initials. #46 is not cited in the text.

Revised
