# Supplementary material for: Defining dual-axis landscape gradients of human influence for studying ecological processes
Source: PLoS One. 2021 Nov 18;16(11):e0252364. doi: 10.1371/journal.pone.0252364 (PMC8601559; doi:10.1371/journal.pone.0252364)
Supplement: Attachment — Submitted filename: Response_to_Reviewers.docx [file pone.0252364.s006.docx]

Dear Janice Bossart,

Thank you for the detailed editorial review of our manuscript. We have made all final changes, and we trust you will agree that all necessary modifications and edits. Below you will find the final round of revisions, with our edits in red.

1. I can appreciate that you prefer to leave the information on your case study species in the three separate locations even though it seems redundant to this reader to be told multiple times that your case study species is the American Robin, that the American Robin is *Turdus migratorious*, and that the American Robin is a widespread generalist found in areas with humans.  At the very least, the 2nd mention of the scientific name is unnecessary since the American Robin was already identified as such the first time.  Please delete that second mention.

Thank you for standing firm on this. We have removed the second reference to the scientific name in line 191. Further, we have removed our description of the species ecology in lines 95 through 96, and it now reads “We demonstrate the utility of our approach in a case study analysis of American robin (*Turdus migratorious*), occupancy.”

2. Lines 410-412.  Incomplete sentence:  “It is important to note, however, that city-specific means in our global analysis which may influence interpretation and comparison of effect size between groups.”  Given you're largely, but not entirely, incorporating what the reviewer literally suggested, I'm not exactly sure what changes are needed.  The sentence would easily make sense by simply removing the 'which'.  That said, some readers less familiar with your analyses might appreciate more complete inclusion of the 'city-specific means differing from the global mean' verbiage, as the reviewer included.

We have removed ‘which’ in order to make the sentence complete. Further, to aid in providing the reader with clarity regarding city-specific means, we have added the following to the text: “city-specific means (i.e., mean effect of landscape on occupancy) in our global analysis”

3. Many corrections are needed in the references

Use lower case in the titles: L495, L502, L507, L512, L537, L648, L698, L711

Journal name should not be all caps: L529, L526, L547

Italicize scientific names: L571, L680

L562. Delete 1st Ecol

L677. Delete (80-) after 'Science'

L585. Lower case 'Framework'

All references have been corrected
